# A Killer Smell: Mold Toxin Destroys Olfactory Cells in Mice

**Published:** 2006-07

**Authors:** Julie Wakefield

Mold seems ubiquitous: it permeates spaces made damp by leaking water lines, faulty roofs, or storm flooding. Although no one contests that its slimy presence is a general nuisance, its related adverse health effects have been the subject of some controversy. Now researchers at Michigan State University’s Center for Integrative Toxicology have found that a toxin produced by the black mold *Stachybotrys chartarum* can damage nerve cells key to the sense of smell, at least in the noses of mice **[*EHP* 114:1099–1107; Islam et al.]**. The study is the first to probe how inhaling black mold toxins affects nasal passages.

Other researchers have previously reported links between *S. chartarum* exposure and human health effects including upper and lower respiratory illnesses. There is also evidence of an association between exposure to fungi in a damp indoor environment and effects such as asthma symptoms in sensitive individuals. However, in a recent Institute of Medicine report, a panel of experts concluded that there is limited or insufficient evidence to determine whether an association exists for other suggested health outcomes such as chronic obstructive pulmonary disease, neuropsychiatric symptoms, skin symptoms, and immune diseases.

The Michigan team found that a single low dose of satratoxin G administered directly into the noses of mice selectively killed sensory neurons involved in detecting odors and sending signals to the olfactory bulbs in the brain. Satratoxins are a type of mycotoxin found in the spores and other parts of *S. chartarum*. The toxins killed the olfactory neurons by apoptosis while apparently leaving bystander cells unharmed. The mice that inhaled the fungal toxins also developed inflammation of the nasal passages and rhinitis (“runny nose” symptoms), as well as milder inflammation of the olfactory bulbs.

It is still unclear how these findings apply to humans exposed to molds. Moreover, before broader health impacts may be assessed, both the amounts of mycotoxins in the air and the nature of human exposure need to be better understood, as do the effects of mold toxins on humans’ sense of smell and nasal inflammation. On first examination, however, these mouse studies suggest that exposure to airborne mold toxins may adversely affect people’s ability to smell. At a minimum, the study raises new questions about the hazards of exposure to black mold in water-damaged buildings.

## Figures and Tables

**Figure:**
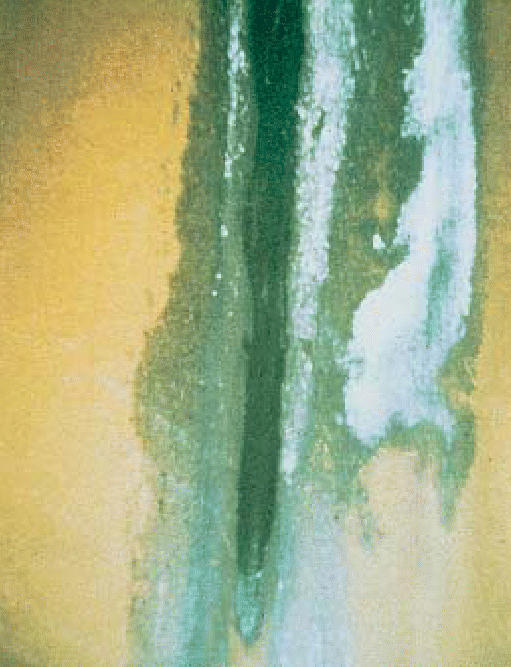
Eau de *Stachybotrys* The mold’s toxin kills olfactory neurons.

